# Hospital administrators as forgotten partners in rare disease care: a call to action by the international hospital federation’s global rare pediatric disease network

**DOI:** 10.1186/s13023-024-03459-5

**Published:** 2024-12-04

**Authors:** Andrea Stoesz, Barbara Joers, Amy Gaviglio

**Affiliations:** 1 Gillette Children’s Specialty Healthcare, 200 University Avenue E., St. Paul, MN 55101 USA; 2 Connetics Consulting, 5737 Standish Avenue, Minneapolis, MN 55417 USA

**Keywords:** Pediatric rare disease, Hospital administration, International

## Abstract

**Background:**

The global public health burden of rare diseases has become an increasingly discussed topic, and its societal impact cannot be overstated. While it may seem counterintuitive to discuss broad healthcare and public health impact in the context of rarity, taken together, over 400 million people worldwide are estimated to live with a rare disease. Over half of people living with a rare disease are children.

Providing robust and comprehensive services to the rare disease community requires coordinated efforts of numerous experts and partners. Globally, there are many initiatives focused on improving the lives of people living with a rare disease. Most of these networks and organizations are region or country based and have historically centered on three focal areas: research; provision of education, support, and/or information; and direct clinical care.

While all these efforts recognize the importance of a coordinated system of partners across a spectrum of disciplines to improve care for the rare disease community, one group has been largely untapped: hospital administrators and leadership. To address this gap, the International Hospital Federation (IHF) convened the Global Rare Pediatric Disease Network (GRPDN), composed of hospital leaders from around the world. To assess how hospital leadership can assist in providing the infrastructure for improving care for patients and families living with a rare disease, the GRPDN created a survey to gather feedback on hospital administrators’ perspectives on needed efforts to improve global rare disease care.

**Results:**

The survey identified five themes: increased public awareness of rare diseases and support for families, diagnostic management and treatment guidelines, lifelong, multidisciplinary care, data and research, and funding.

**Conclusions:**

Until recently, hospital leadership has been an untapped partner in addressing challenges faced by rare disease patients, and they are uniquely positioned to bridge existing gaps. The GRPDN will continue to focus on identifying practical strategies that hospital leaders—regardless of resource level—can implement to improve care for children living with a rare disease.

## Background

The global public health burden of rare diseases has become an increasingly discussed topic, and its societal impact cannot be overstated. While it may seem counterintuitive to discuss broad healthcare and public health impact in the context of rarity, taken together, over 400 million people worldwide are estimated to live with a rare disease. Over half of people living with a rare disease are children, and of those, approximately 30% will die before the age of five due to lengthy diagnostic and therapeutic odysseys [1].

Patients living with a rare disease face diagnostic, prognostic, and therapeutic odysseys. Owing to a general lack of awareness of rare disease and availability and accessibility of appropriate testing and specialists, the diagnostic odyssey can be lengthy and can lead to prolonged periods of stress for patients and their families as they navigate a complex healthcare system and are managed by multiple specialists [2]. Once a rare disease is successfully diagnosed, the absence of treatment and management guidelines for many rare diseases is equally challenging. Only about 5% of rare diseases have an approved treatment available, leaving the vast majority of patients without any approved treatment options [3, 4]. This means that patients must rely on symptomatic management rather than disease-modifying therapeutic options.

The global impact of rare diseases is not limited to the clinical and social issues directly related to the rare disease itself. The diagnostic and treatment uncertainties that are hallmarks of rare diseases have been shown to also have a detrimental effect on the health, psychosocial, and economic well-being of rare disease patients and their families [5, 6]. Understanding this, in 2021, the United Nations General Assembly adopted a resolution related to the challenges that people living with a rare disease and their families face and called for member nations to recognize the rare disease community as a marginalized group that should be given particular consideration during healthcare planning [7].

From a hospital administration perspective, it is costly and logistically difficult to maintain the availability of highly specific expertise, appropriate diagnostic and treatment equipment and materials needed to care for rare disease patients. Due to these high costs, many hospitals—particularly those in underfunded areas—find it difficult to adequately invest in the staff and resources needed to care for rare disease patients. This has led to the concentration of such resources, often in urban areas. This creates access issues for patients living in underfunded areas, adding an additional layer of burden and emotional and financial stress. Understanding resource limitations that hospital administrative leaders must contend with, it is important to prioritize and provide guidance for ways this group can collaboratively address the needs of the rare disease community.

Providing robust and comprehensive services to the rare disease community requires coordinated efforts of numerous experts and partners. Globally, there are many initiatives focusing on improving the lives of people living with a rare disease. Most of these networks and organizations are region or country based and have historically centered on three focal areas: research; provision of education, support, and/or information; and direct clinical care.

While all these efforts recognize the importance of a coordinated system of partners across a spectrum of disciplines to improve care for the rare disease community, one group has been largely untapped: hospital administrators and leadership. Owing to the documented need for coordinated care across healthcare systems, hospitals, and clinics, the exclusion of hospital administrators as key partners is concerning [8]. Hospital leaders must be integrated into rare disease work to help build the collaborative and innovative infrastructure needed to support children and families living with a rare disease and contribute to the robust implementation of existing research, education, support, and clinical care efforts.

## The global rare pediatric disease network

To address this gap, the International Hospital Federation (IHF) convened the Global Rare Pediatric Disease Network (GRPDN), composed of hospital leaders from around the world. The purpose of the GRPDN is to develop and maintain a network of IHF member facilities that provide care for children living with a rare disease to share ideas and learnings at the hospital administrator and leadership levels.

Hospitals currently participating in the GRPDN represent Belgium, Canada, England, Saudi Arabia, Singapore, Spain, United States, and Zambia. The GRPDN is open to other IHF members, with several hospitals indicating interest in joining.

To assess how hospital leadership can assist in providing the infrastructure for improving care for patients and families living with a rare disease, the GRPDN created a survey to gather feedback on hospital administrators’ perspectives on needed efforts to improve global rare disease care.

## Methods

A subgroup of GRPDN members drafted a 25-question survey. The survey was piloted by individuals within the GRPDN as well as by external individuals with international expertise in rare diseases and survey development. The survey’s validity was assessed through a multi-step process. First, it was reviewed by experts to assess content validity and whether the questions effectively captured the topic under investigation. The survey was also assessed by an individual with competency in survey research to ensure questions were not leading or confusing. Feedback from these individuals was incorporated. The survey was then shared with members of the GRPDN to assess consistency in responses across potential participants.

Survey data were collected and managed using REDCap electronic data capture tools hosted at Gillette Children’s Specialty Healthcare, a GRPDN member. The survey was open for approximately three months. Eight surveys were completed by facilities in Belgium, Canada, England, Saudi Arabia, Spain, Taiwan, and the United States. Surveys were completed by individuals in clinical leadership positions within their respective institutions.

The survey asked two qualitative questions with open-ended responses. Using thematic analysis, two independent reviewers analyzed the qualitative data and identified cross-cutting themes. Themes were not pre-identified. Rather, survey responses were reviewed in their entirety and then reviewed again for themes. Reviewers compared notes and identified themes. Due to the relatively small dataset and heterogeneity of the responses, there were only minor differences in the identified themes. Differences were reconciled by collaboratively reviewing the data to assure explicit and implicit meanings were interpreted in the same manner.

The two qualitative questions were:

*If you were to prioritize three actions that would positively impact outcomes for patients living with a rare disease, what would they be?* and,

As leaders, [IHF] encourages agencies, governments, and others to better understand the

*needs of children living with a rare disease. What advocacy, policy, and/or improvement ideas would you like IHF to consider in our efforts?*

## Results

**Fig. 1:**
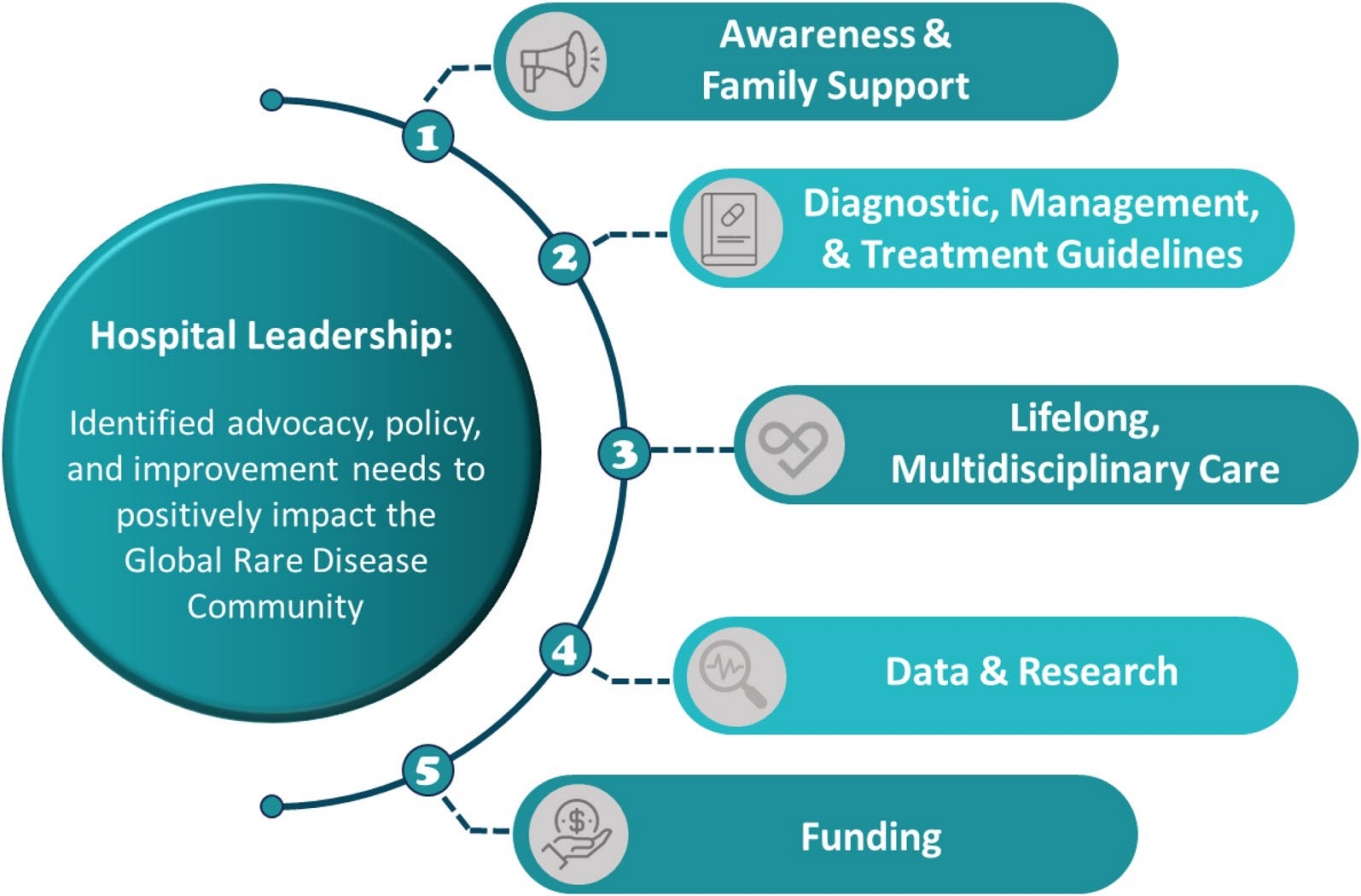
Policy, advocacy, and improvement themes identified by hospital leaders

Survey reviewers identified five themes across the two qualitative questions: (1) increased public awareness of rare diseases and support for families, (2) diagnostic management and treatment guidelines, (3) lifelong, multidisciplinary care, (4) data and research, and (5) funding (Fig. 1).

Themes identified by each participating hospital are shown in Table 1.

**Table 1:** Summary of qualitative survey themes identified by each respondent

Hospital	Themes
1	Increased public awareness and support for families, diagnostic management and treatment guidelines, data and research
2	Increased public awareness and support for families, diagnostic management and treatment guidelines, data and research
3	Lifelong, multidisciplinary care
4	Increased public awareness and support for families, data and research, funding
5	Increased public awareness and support for families, data and research, lifelong, multidisciplinary care, funding
6	Increased public awareness and support for families, funding
7	Lifelong, multidisciplinary care
8	Lifelong, multidisciplinary care, data and research, funding

## Discussion

The identified themes have long been considered needed components and point to larger systemic challenges that have hampered care for rare disease patients, their families, and those who provide care for them. What is unique to this work, however, is the focus on the role of hospital leadership, which has been an oft-ignored partner. While hospital administrators cannot solve these issues on their own, there are several actions that leaders can take to positively impact children living with a rare disease and their families. Each theme is discussed in more detail below with this context in mind.

### Increased public awareness of rare diseases and support for patients and families

#### Background

Despite the wide-reaching impacts of rare diseases, there remains a general lack of awareness of these conditions and their unique and cross-cutting challenges. This lack of awareness is found among the general public, healthcare providers and administrators, as well as policymakers [9–12].

Increasing awareness of rare diseases and their diagnoses and management is not simply an exercise in improving general knowledge. Building awareness is foundational to the future of rare diseases in several ways. First, it can help create a more inclusive society by reducing the stigma and isolation many individuals and families with rare diseases experience. Second, awareness can help improve access to appropriate medical care. Indeed, a lack of awareness of rare diseases is likely contributing to the lengthy diagnostic odyssey and mismanagement experienced by many rare disease patients and families. And finally, raising awareness can help encourage novel research and increase the developmental pipeline of new treatments for rare diseases.

#### Call to action for hospital leaders

Hospital administrators are an important stakeholder in addressing this challenge. Creating a culture of rare disease awareness and advocacy at the institution and community level can result in numerous positive downstream effects.

**Fig. 2:**
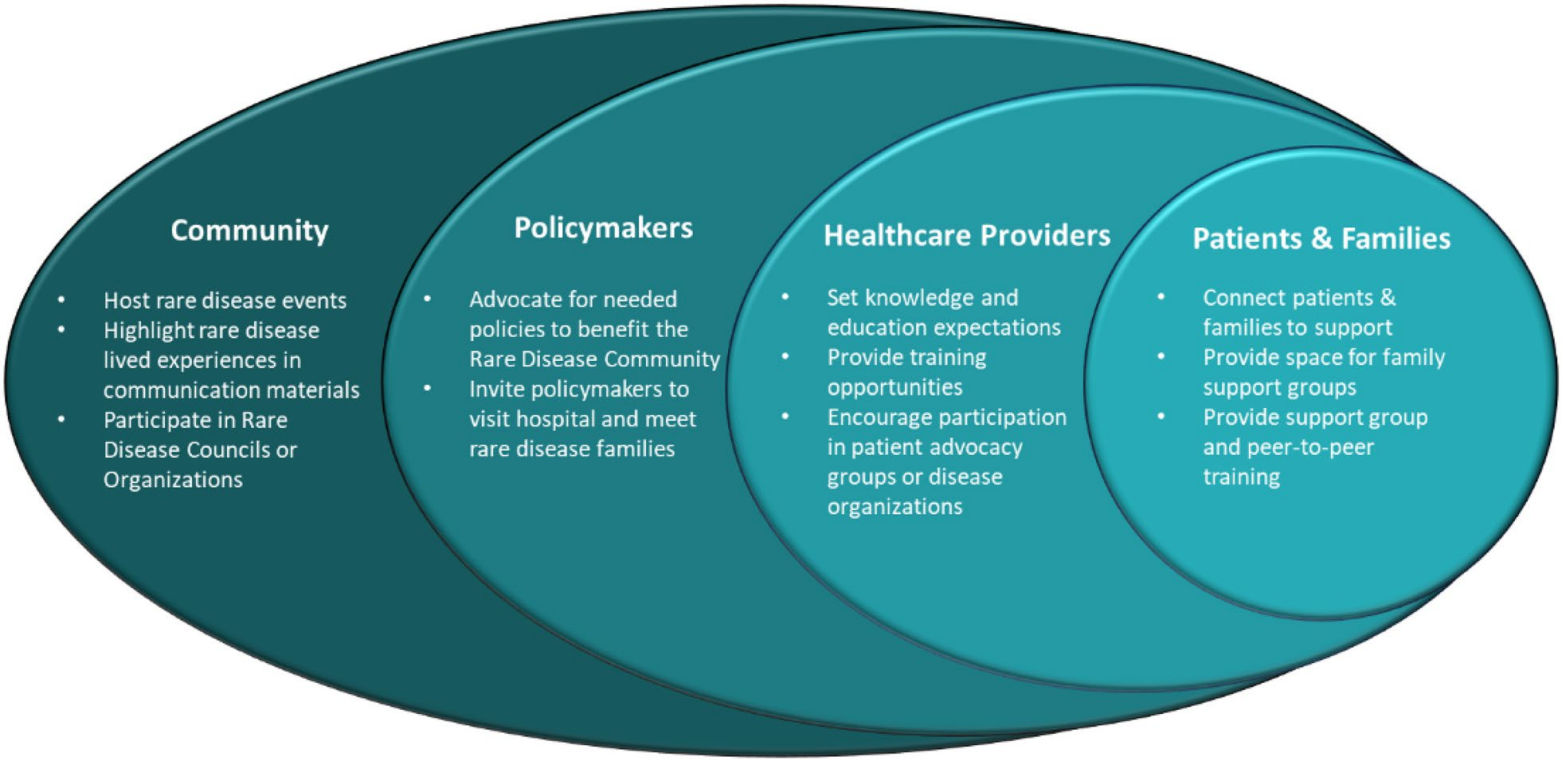
Actions for hospital leaders to take to improve awareness at various levels

Hospital leaders have an opportunity to improve awareness at various levels (Fig. 2).

Because hospital leaders play a unique role in community education, they can greatly contribute to raising awareness of the existence and needs of rare diseases. Improved awareness efforts could encompass simple actions, such as including the lived experience of rare disease families in communication materials or collaborating with local patient advocacy groups or rare disease councils to raise awareness and advocate for policy changes that benefit the rare disease community.

Caregivers are an important part of each patient’s care team. For patients to receive optimal care, caregivers’ mental health and wellbeing must be supported. Hospital leaders can support caregiver and patient wellbeing by connecting patients and families to community-based resources such as patient advocacy and support groups. In jurisdictions where patient advocacy groups may not exist, hospital leaders can donate space to patients and families who want to lead a support group or help provide family-to-family support training. For many rare disease families, a sense of community is seen as a much-needed form of disease management.

### Diagnostic management and treatment guidelines

#### Background

It is well known that people living with a rare disease may wait years or even decades until they receive an accurate diagnosis. Shortening the diagnostic odyssey is a major goal of those involved with the rare disease community. Numerous initiatives toward this end have focused on educational efforts of non-genetics providers, utilizing checklists or other mechanisms to highlight clinical red flags that may point to a possible rare disease diagnosis [13, 14].

Improved training and education of clinicians are becoming increasingly essential to addressing the diagnostic odyssey as there is a significant gap between needed genetic services and the current capacity of the genetics workforce. Recent reports suggest that there are less than two geneticists per one million people in the United States, and this lack of workforce most certainly exacerbates systemic factors leading to significant diagnostic delays [15].

Though much work has, understandably, focused on the diagnostic odyssey, it is also important to recognize that a rare disease diagnosis often starts the family on yet another difficult journey—one that may involve uncertain management guidelines or treatment needs and availability. Over 90% of rare diseases lack an FDA-approved treatment [16]. This means that rare disease families are often faced with less-than-ideal therapeutic options with unclear management needs.

Development and implementation of treatment and management guidelines for rare diseases presents a difficult challenge for clinicians. In many cases, the rarity of the diseases and the geographical spread of patients make it difficult for any single center to establish the expertise needed to develop evidence-based guidance.

Even when guidelines do exist, there are varying levels of implementation and use by clinicians. Gittus, et al., explored challenges and facilitators to rare disease guideline implementation and found that awareness and familiarity with the recommended treatment guidelines is the most common reason why treatment guidelines are not implemented by healthcare providers [17].

#### Call to action for hospital leaders

Understanding that increasing and diversifying the genetics workforce will take significant time, there have been increasing calls to educate and train non-genetics providers to properly order genetic and genomic testing and effectively communicate these results to patients. However, very few programs have been implemented to provide this training to non-genetics providers within a specific healthcare environment. This is an area where hospital leaders can invest to improve patient care.

Training other providers to recognize potential rare diseases, understanding the best testing methodology to elucidate the diagnosis, and ensuring clear and appropriate referrals to genetics can not only decrease the diagnostic odyssey but can make genetic testing and care generally more accessible. Additionally, hospital leaders can assure that providers have sufficient time to conduct research and stay up to date with current treatment guidelines for rare diseases.

Hospital leaders can also encourage cross-institution collaboration on the development of guidelines for rare diseases. This can be done through providing the infrastructure (IT, grants, or otherwise) for multi-center studies and data sharing. Likewise, health leaders may be best equipped to address reported organizational-level barriers to the implementation of clinical guidelines—such as the lack of time allotted for research and reading, time for clinical consultations, and a shortage of resources such as access to newer electronic applications and alerts or integration of guidelines into the electronic medical record [18].

### Lifelong, multidisciplinary care

#### Background

Rare diseases are highly complex, multi-systemic conditions, often requiring multiple specialists and a coordinated system of care [19].However, because the number of patients with a specific rare disease is small, it can be difficult to determine how best to organize care. Historically, a multidisciplinary model of rare disease care has been encouraged, and more recently, a global network of care has been established [20].

While multidisciplinary models have often been limited to addressing the physical aspects of the disease, there are increased calls for these care models to also include psychological support, ancillary care for daily living, and inclusion of the patient and family as experts by lived experience [21, 22].

A survey by the European Organisation for Rare Diseases found that living with a rare disease or caring for a loved one living with a rare disease has a significant impact on social and family life, leading to feelings of isolation. The survey also found that rare disease patients’ mental health is worse compared to the general population [23].

Additionally, children with special healthcare needs—such as those with a rare disease—may be at higher risk of experiencing adverse childhood events and of experiencing medical trauma [24].

Medical trauma can impact both patients and their caregivers and may happen repeatedly. For rare disease patients and families, potentially traumatic experiences might include experiencing the loss of a child or almost losing a child, enduring painful medical procedures, navigating complex medical and insurance coverage systems, difficult, uncertain, or painful treatment plans, difficulty in accessing care, guilt or stigma, provider bias, or minimization of concerns by a healthcare provider.

An important part of addressing medical trauma is assuring high-quality patient and family experiences. And increasingly, care coordination for children with medically complex conditions is viewed as an essential element to reduce the fragmented care that rare disease patients so often experience [25].

#### Call to action

Hospital leaders should invest in assuring access to multidisciplinary clinics as well as care coordination. Care coordination models have been shown to be effective for patients with cancer or a chronic condition [26, 27]. Increasingly, care coordination is being viewed as essential in supporting patients with rare diseases due to the complexity of the conditions and the otherwise disparate care received. Care coordination should also include proactive and early transition planning for when pediatric patients age out of the hospital system. Transition to adulthood for rare diseases is becoming increasingly important as more children with rare diseases are surviving into adulthood. Specific considerations on disparities seen in transition also need to be examined as gaps in transitional care can undermine previous gains and efforts made during childhood [28–30]. Hospital leaders can support transition care and planning by advocating for and assessing payment models that reimburse for transition services. Additionally, hospital leaders can begin to generate data to inform future policy and payment models for these services.

Finally, hospital leaders have a significant part to play in minimizing medical trauma for rare disease patients and their caregivers. Specific examples include engaging the patient and their family in care planning, training all staff in trauma-informed care, screening patients for trauma, and using trauma-specific intervention where appropriate. These simple actions will help ensure that hospitals do not inadvertently add harm to patients and families living with a rare disease.

### Data and research

#### Background

Lack of robust rare disease data and research is a well-documented challenge, fueled by operational, regulatory, and economic barriers [31]. Managing and processing health data as it pertains to rare disease is a systemic problem that requires a systemic and global solution. The post-pandemic era has afforded numerous opportunities in innovative use of health informatics and data-driven care rooted in the use of real-world data [32].

Rare diseases are particularly susceptible to gaps in data because of their low prevalence and heterogenous nature. Likewise, many electronic health records have not been set up to collect and/or leverage needed data in discrete ways to allow for the analytics and assessments needed for natural history or treatment efficacy studies.

There is an ongoing need to move rare disease data towards the FAIR principle (Findable, Accessible, Interoperable, and Reusable) while also acknowledging the inherent sensitivity and potential re-identifiability of data associated with rare diseases [33]. But wide variations continue to exist in how data is collected, formatted, and stored across facilities caring for individuals living with rare diseases. Though much work continues in this realm with the use of Common Data Elements, terminology standards, and aggregation platforms, a truly interoperable rare disease data network is still lacking.

#### Call to action for hospital leaders

Data management in rare disease requires numerous partners beyond standard informaticians. Clinicians caring for rare disease patients and families will need enhanced skills in data management and an understanding of the negative impacts of siloed data collection approaches. Hospital leaders can upskill their workforce by providing training on data management and rare disease data needs to staff while also increasing subject matter expertise in health informatics within their systems.

Likewise, encouraging use of standard terminology and Common Data Elements within the hospital’s electronic health record and creating a culture of standardized nomenclature will assist in future data analytic needs, and is particularly important when considering future use of artificial intelligence-based analytics and modeling [34].

Ultimately, hospital leaders need to appreciate the value of standardized and interoperable data in the rare disease space and invest in mature electronic health record systems and a data-savvy workforce as an integral part of their care model for rare diseases.

### Funding

#### Background

Due to the geographic dispersion of rare disease patients and the previously underrecognized impact of rare diseases, there has been a global underinvestment in the rare disease community. This has resulted in a lack of public funding for rare diseases as well as a lack of policy strategies to address the challenges that impact people living with a rare disease [35]. This has led to hospitals and rare disease patients and their families bearing a significant amount of the economic burden related to rare disease. For example, one recent study found that lack of treatment for a rare disease resulted in a 21% increase in cost of care per patient [36]. The same study estimated the societal cost of rare diseases—in the United States alone—to be between $7.2 trillion and $8.6 trillion each year.

While there remains a paucity of treatment options for rare diseases, even when treatment is available, it is often not accessible. However, aggressive early detection and diagnosis of rare disease as well as early access to treatment can ultimately reduce costs over a patient’s lifetime [37]. Rare disease treatments, especially novel gene and cellular therapies, can be prohibitively expensive for patients, costing millions of dollars per dose and are not always covered by insurance providers. This means that hospitals may be left in the difficult position of having to choose between providing life-altering care while absorbing an expensive financial loss or withholding needed treatment.

#### Call to action for hospital leaders

There must be greater public investment in rare disease care to assure that patients have access to the care they need, when they need it, where they need it. Though larger system funding issues may be out of hospital leadership control, hospital leaders can advocate for solutions that lower costs for patients or ensure all-encompassing coverage of both clinical trial participation and approved treatment administration.

For example, leaders can advocate for policy solutions to spur quicker development of rare disease treatments, such as incentives for pharmaceutical companies to invest in rare disease drug development or extension of market exclusivity of a treatment. Leaders could also advocate for increased governmental investment in rare disease research as well as payment models that reimburse for comprehensive, multidisciplinary care.

Private–public partnerships are likely going to be needed to sustain appropriate care for the rare disease community and assist in more seamless transitions from bench to bedside. Hospital leaders can begin forming these partnerships and participating in broad coalitions to bring funding and investments to this space.

## Study limitations

A limitation of this study was that a small number of hospitals responded to the survey. This was likely due, at least in part, to difficulties pulling requested data and the survey only being available in English and only available to certain IHF members.

## Conclusion

The challenges faced by rare disease patients are well-documented. There have been enormous efforts on the national and international levels to identify and begin to address these issues. Until recently, hospital leadership has been an untapped partner in this work, and they are uniquely positioned to bridge existing gaps.

Hospital leaders play an important role in the community—whether by connecting patients to advocacy or support groups, or by helping raise awareness of rare diseases with the public and policymakers.

Children’s hospitals also serve as vital hubs for education and training, equipping healthcare professionals with the knowledge and skills required to accurately diagnose and effectively manage rare diseases. Better education, in turn, leads to earlier interventions, improved patient outcomes, and reduced healthcare costs over the long term.

The GRPDN will continue to focus on identifying practical strategies that hospital leaders—regardless of resource level—can implement to improve care for children living with a rare disease. The GRPDN will also consider how to best share these strategies with hospitals across the globe.

Though the GRPDN’s survey focused on hospitals, similar solutions could be implemented by healthcare delivery systems. By harnessing the collective power of healthcare institutions, patients, policymakers, and other partners, we can forge a brighter path forward for people living with rare diseases. And, as precision medicine becomes increasingly accessible and underlying genetic causes separate common diseases into separate disease categories, these challenges will also apply to nearly all diseases and conditions.

Without solving these challenges for the relatively small, rare disease population, doing so on a larger scale will be difficult. The time is ripe to unlock the full potential of hospitals in the pursuit of improved care and outcomes for people living with a rare disease.

## Data Availability

The deidentified dataset analyzed for this study is available from the corresponding author on reasonable request. Survey participants were advised that their deidentified responses would only be shared upon their approval.
